# Highly anomalous accumulation rates of C and N recorded by a relic, free-floating peatland in Central Italy

**DOI:** 10.1038/srep43040

**Published:** 2017-02-23

**Authors:** Claudio Zaccone, Daniela Lobianco, William Shotyk, Claudio Ciavatta, Peter G. Appleby, Elisabetta Brugiapaglia, Laura Casella, Teodoro M. Miano, Valeria D’Orazio

**Affiliations:** 1 Department of the Sciences of Agriculture, Food and Environment, University of Foggia, via Napoli 25, 71122 Foggia, Italy; 2 Department of Soil, Plant and Food Sciences, University of Bari “Aldo Moro”, via Amendola 165/A, 70126 Bari, Italy; 3 Department of Renewable Resources, University of Alberta, 348B South Academic Building, T6G 2H1, Edmonton, Canada; 4 Department of Agricultural Sciences, Alma Mater Studiorum University of Bologna, viale Fanin 40, 40127 Bologna, Italy; 5 Department of Mathematical Sciences, University of Liverpool, Liverpool L69 3BX, United Kingdom; 6 Department of Agricultural, Environmental and Food Sciences, University of Molise, via Francesco De Sanctis, 86100 Campobasso, Italy; 7 Italian National Institute for Environmental Protection and Research, via Vitaliano Brancati 60, 00144 Roma, Italy

## Abstract

Floating islands mysteriously moving around on lakes were described by several Latin authors almost two millennia ago. These fascinating ecosystems, known as free-floating mires, have been extensively investigated from ecological, hydrological and management points of view, but there have been no detailed studies of their rates of accumulation of organic matter (OM), organic carbon (OC) and total nitrogen (TN). We have collected a peat core 4 m long from the free-floating island of Posta Fibreno, a relic mire in Central Italy. This is the thickest accumulation of peat ever found in a free-floating mire, yet it has formed during the past seven centuries and represents the greatest accumulation rates, at both decadal and centennial timescale, of OM (0.63 *vs.* 0.37 kg/m^2^/yr), OC (0.28 *vs.* 0.18 kg/m^2^/yr) and TN (3.7 *vs.* 6.1 g/m^2^/yr) ever reported for coeval peatlands. The anomalously high accretion rates, obtained using ^14^C age dating, were confirmed using ^210^Pb and ^137^Cs: these show that the top 2 m of *Sphagnum*-peat has accumulated in only ~100 years. As an environmental archive, Posta Fibreno offers a temporal resolution which is 10x greater than any terrestrial peat bog, and promises to provide new insight into environmental changes occurring during the Anthropocene.

Floating mires are peculiar environments consisting of emergent vegetation rooted in highly organic buoyant mats that rise and fall with changes in water level. The whole floating mass is generally divided into a mat root zone and an underlying mat peat layer^1^; the release of gases due to the decomposition of organic matter (OM) is generally the main cause of buoyancy^2^. Floating mires are distributed world-wide; they range from small, free-floating islands to extensive, stationary, vegetated mats which may cover hundreds of hectares of water^3^. Large areas of floating marsh occur along rivers and lakes in Africa, the Danube delta in Romania, the Amazon River in South America, and in the Mississippi delta in USA, whereas smaller areas occur also in The Netherlands, Australia and Canada^4^,^5^,^6^. Their thickness and buoyancy can vary considerably and depend upon both below-ground biomass allocation and morphology of the constituent plant species^6^, as well as on abiotic factors such as salinity and hydrology^7^. Free-floating mires (able to support the weight of at least one person) normally show a thickness ranging from 40 to maximum 200–250 cm. Studying floating mats from the Louisiana delta plain, Swarzenski *et al*.^4^ found a relatively consistent thickness among mats of different ages, thus suggesting that they may reach a maximum thickness beyond which vertical accretion is balanced by loss of OM through decomposition^8^.

The fascinating sight of an island floating and moving mysteriously on a lake naturally intrigued people from time immemorial. In fact, this phenomenon was already described, among others, by Seneca (4 bc-ad 65) in his *Naturales quaestiones*, and by Pliny the Elder (ad 23–79) in his *Naturalis historia*. Latin authors have left us suggestive descriptions of floating islands, including those in Lacus Cutiliensis (now Lago di Paterno) and in Lacus Vadimonis (now a marshy pond), reporting details about their size and shape, buoyancy and vegetation. But the status of “flotant” has been defined transitory^9^; in fact, these small isles often disappear, in most of the cases because a transition from free-floating islands to firm land during decades, or their sinking, is likely to occur^10^. That is probably why none of the free-floating islands described by Latin authors as occurring in several lakes (*e.g.*, Cutiliensis, Mutinensis, Statoniensis, Lydia Calaminae, Vadimonis) are extant today.

Several papers have been published about natural floating islands, mainly focusing on hydrological^4^,^5^ and ecological^5^,^7^,^11^ aspects, as well as on their management^9^,^12^ or utilization to improve water quality. A fascinating and valuable review of the writings on floating islands, including their distribution, modes of formation, composition, buoyancy and movements, and role in human society (control and management, habitation, artificial islands, and mythological and esoteric aspects) was provided by Van Duzer^13^, who begins his treatise with a reproduction and translation of a 1711 dissertation by the German priest Georg Christophy Munz on floating islands (*Exercitatio academica de insulis natantibus*). At the same time, numerous questions are still unanswered, including the role of free-floating islands in the global C and N cycles.

Accumulation rates (AR) of both organic C (OC) and total N (TN) have been extensively investigated along the bog-to-fen gradient; peatlands accumulate 20–30 gC/m^2^/yr over the long-term^14^,^15^,^16^, with bogs generally showing higher rates of C accumulation than fens^17^, and 0.5–0.9 gN/m^2^/yr (refs 18 and 19), with fens having higher rates of N accumulation than bogs^19^,^20^. In contrast, there have been no studies regarding the long-term AR of OC and TN in free-floating systems. A few studies focusing on net ecosystem exchange measures on a short term timescale (daily, seasonal, annual) and/or in laboratory conditions demonstrated that floating mats may represent a net C source^21^,^22^.

Here we provide the first, high resolution record of OC and TN AR obtained from a 4 m-deep free-floating mire. The floating island of Posta Fibreno is one of the few surviving in southern Europe. With the exception of a 3 m-deep floating mat (mainly rhizomes of *Scirpus californicus*) located in a collapsed crater on Easter Island^23^, there are no reports in the contemporary scientific literature about free-floating mires having a comparable thickness. The latitude and the sub-Mediterranean climate of Posta Fibreno make it an ecological rarity. Investigating this unique environment, we provide new insights toward understanding OM biogeochemistry in free-floating systems, as well as the first inventories, chronologies and AR of OC and TN for this ecosystem. Finally, we assess the potential of this peculiar ecosystem to be used as high-resolution archive of environmental changes and human activity impact for the past centuries.

## “La Rota”, A Fascinating Free-floating Mire

**Figure 1:**
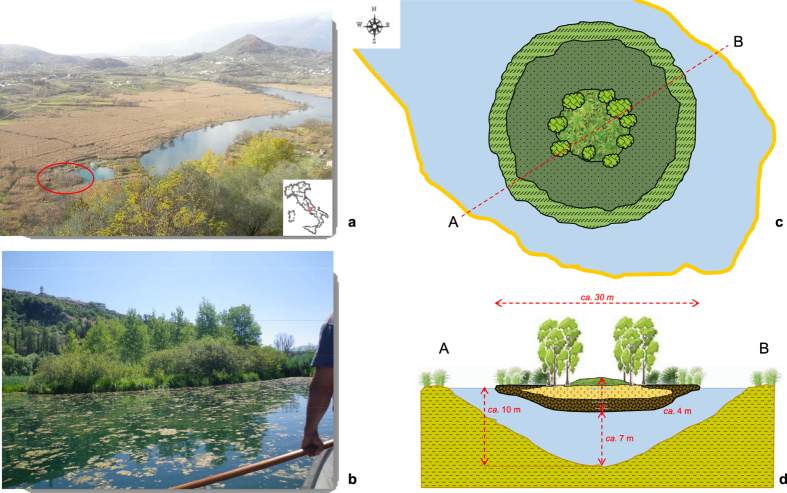
The Posta Fibreno free-floating island (“la Rota”). (**a**) View of the Posta Fibreno lake, with a red circle surrounding the free-floating island (photo by C.Z.). The map in the bottom right corner was created using QGIS v. 2.14 software (http://www.qgis.org/it/site/). (**b**) the Posta Fibreno free-floating island (photo by C.Z.). (**c,d**) Schematic representation of the studied free-floating mire (drawn by C.Z.). The 400-cm deep peat profile was collected from the central domed area of the floating island.

The protected area of “Lake of Posta Fibreno” (Supplementary Information) represents a refuge site for boreal species^24^. Here, a free-floating island known as “La Rota”, a relic mire in Central Italy, moves erratically on the water surface of a submerged doline (sinkhole) annexed to the easternmost edge of the lake (Fig. 1a,b). This island migrates as a consequence of either the wind or currents induced by subsurface springs. The floating island has a diameter of *~*30 m, a submerged thickness of almost 3 m, and the vegetation is arranged in concentric belts, from the *Carex paniculata* palisade to the *Sphagnum palustre* center, the latter surrounded by *Populus tremula* trees which have appeared in the last twenty years (Fig. 1c,d). Here, some of the southernmost European populations of *S. palustr*e occur^25^.

At Posta Fibreno, the deep water layer below the base of the island (~7 m; Fig. 1d) probably prevented the transition from free-floating island to firm land, thus allowing it to wander on the surface of the lake for centuries.

**Figure 2:**
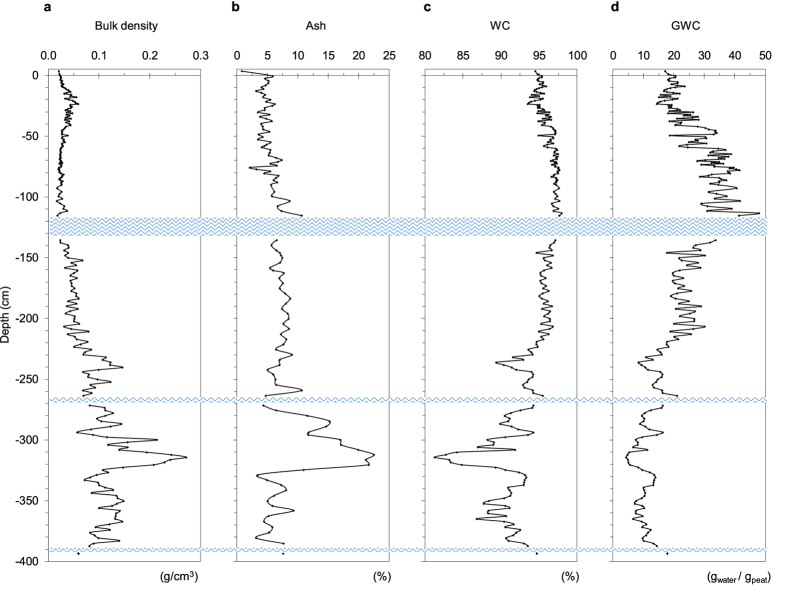
Variation of physical properties of peat with depth. The figure illustrates the variation of bulk density (**a**), ash content (**b**), water content (WC) (**c**) and gravimetric water content (GWC) (**d**) trend throughout the 400-cm deep peat profile. Blue wavy lines represent water lenses occurring throughout the profile. More details are reported in Supplementary Information.

The top 220–230 cm of this free-floating mire consists almost exclusively of *S. palustre* moss, with stems and leaves of Graminaceae and Cyperaceae species that become more abundant between 100 and 230 cm; below this depth, silty peat rich in stems and leaves of Graminaceae and Cyperaceae species, with abundant roots and limited (<1%) *Sphagnum*, occurs (Supplementary Figs S1 and S2). The top 220 cm of the island also shows completely different physical (density, ash content, water content) and chemical (pH, EC) properties compared to the bottom 180 cm of depth (Fig. 2; Supplementary Table S1 and Fig. S3). Further details are provided in the Supplementary Information.

Three thin water layers occur at depths of 117–135 cm, 267–270 cm and 388–392 cm (Supplementary Fig. S1); replicate cores showed that these discontinuities were not created during coring.

### OC and TN inventory and corresponding short and long-term AR

**Figure 3:**
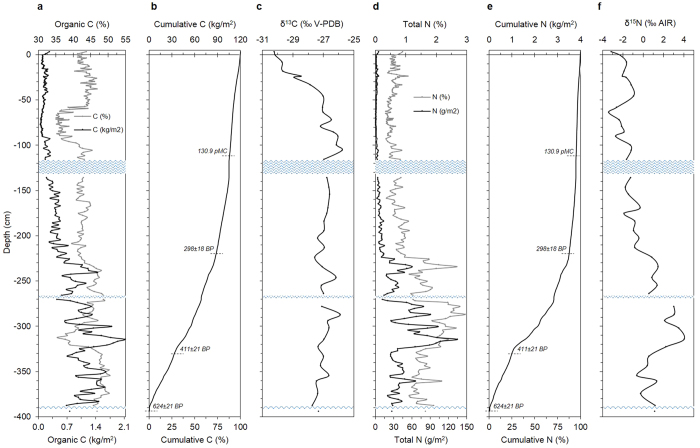
Carbon and nitrogen distribution with depth. The figure illustrates the variation of total and cumulative concentration, as well as of its isotope signature, of both organic C (**a–c**) and total N (**d–f**) throughout the 400-cm deep peat profile. Blue wavy lines represent water lenses occurring throughout the profile. In panels (b) and (e), selected conventional ^14^C age dates are reported (for details, see Supplementary Table S4).

Organic C concentrations along the profile range between 35 and 50%; with the exception of two zones, *i.e.*, between 60–90 cm and 300–325 cm, OC concentrations are quite constant throughout the profile (avg., 43 ± 4%) (Fig. 3; Supplementary Table S1). At the same time, the average mass of OC stored per unit of surface strongly increases with depth (*i.e.*, 0.13 ± 0.05 kg/m^2^ in the top 100 cm, 0.37 ± 0.11 kg/m^2^ between 100 and 200 cm; 0.84 ± 0.32 kg/m^2^ between 200 and 300 cm, and 1.26 ± 0.34 kg/m^2^ down to 400 cm of depth) (Fig. 3a). This results in a cumulative OC of 119 kg/m^2^ of mire surface (corresponding to 245 kgOM/m^2^), 50% of which stored in the bottom 110 cm; in contrast, the top 100 cm account for 11% of the OC accumulated in the whole profile (Fig. 3b). The δ^13^C signature increases almost linearly throughout the top 110 cm (from −30.2 to −25.8‰), whereas it remains around −27.0 ± 0.4‰ along the bottom 290 cm of depth (Fig. 3c). Less negative δ^13^C values detected below 110 cm of depth suggest the occurrence of a more decomposed peat, as OM decay generally results in a ^13^C-enriched residual material as bacteria preferentially metabolize the lighter, ^12^C-rich fraction.

Total N concentration (avg., 1.0 ± 0.6%) increases with depth, showing values almost always <1% (avg., 0.6 ± 0.2%) throughout the first 220 cm, and much higher in the remaining 180 cm (avg., 1.8 ± 0.5%), with maximum concentrations ranging between 2.1 and 2.9% at 272–321 cm of depth (Fig. 3; Supplementary Table S1). As for OC, the average mass of TN stored per unit of surface area also increases with depth (*i.e.*, 1.7 ± 0.9 g/m^2^ in the top 100 cm, 6.2 ± 2.7 g/m^2^ between 100 and 200 cm; 34.2 ± 23.2 g/m^2^ between 200 and 300 cm, and 48.8 ± 26.8 g/m^2^ down to 400 cm of depth) (Fig. 3d). This results in a cumulative TN of 4.1 kg/m^2^ of mire surface, 50% of which stored in the bottom 100 cm, whereas the top 100 cm account for less than 5% of the TN accumulated in the whole profile (Fig. 3e). The δ^15^N shows values ranging from −3.4 to + 4.0‰ (Fig. 3f; Supplementary Table S1). The significantly higher δ^15^N values recorded below 200 cm of depth underline, besides a change of the main peat-forming species, a preferential loss of ^14^N molecules through kinetic fractionation during biological transformation^26^, and thus reflect a greater degree of decomposition^26^,^27^,^28^.

**Figure 4:**
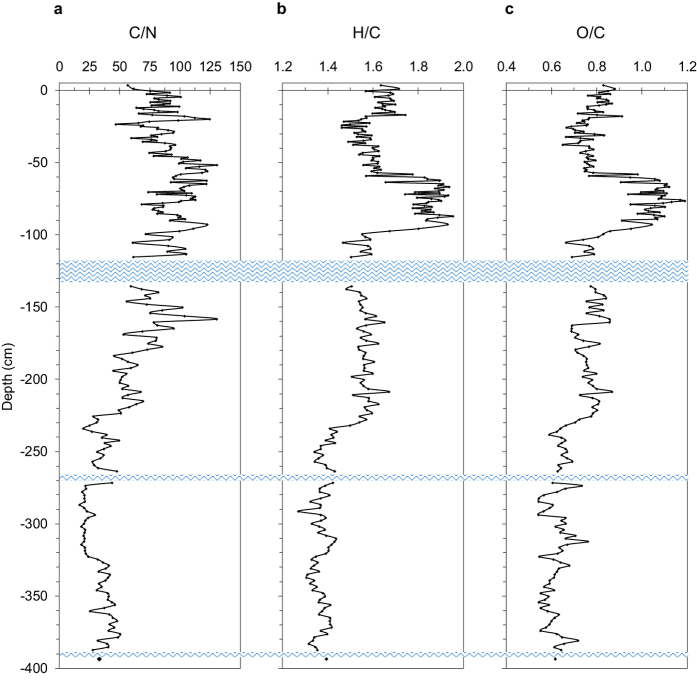
Main atomic ratios variability with depth. The figure illustrates the trend of C/N (**a**), H/C (**b**) and O/C (**c**) throughout the 400-cm deep peat profile. Blue wavy lines represent water lenses occurring throughout the profile.

Peat OM accumulated throughout the free-floating island profile is also characterized by a completely different degree of humification. Here, C/N, H/C and O/C ratios, which are positively correlated among each other (*p* < 0.001) and negatively with density and δ^15^N (*p* < 0.001) (Supplementary Fig. S3 and Table S2), generally decrease with depth, suggesting the occurrence of more humified materials below 220 cm of depth (Fig. 4). The occurrence of very well preserved (almost undecomposed) *Sphagnum* material in the top 100 cm, and a more humified OM below *ca.* 200 cm of depth, is very clear also from FT-IR spectra (Supplementary Fig. S3 and Table S4). In fact, peat samples selected from the first 100 cm are characterized by a number of absorption bands that typically occur in the top, few centimeter-deep layers of *Sphagnum*-dominated peatlands^29^,^30^, whereas below 200 cm of depth, FT-IR spectra suggest that OM undergone a more pronounced decomposition process (Supplementary Information).

Several authors reported increases of peat thickness due to the *Sphagnum* growth in the order of 0.07–0.60 kg/m^2^/yr, corresponding to 0.03–0.24 kgC/m^2^/yr, although these values refer to different environmental conditions as well as laboratory incubations^22^,^31^,^32^. Here, using radiocarbon (Supplementary Table S3) and ^210^Pb (Supplementary Tables S5 and S6) age dates, we estimated average peat AR on both short and long-term timescales (Table 1). The top 100 cm, corresponding to the last ~50 yr of peat formation (AD 1964–2012), show an average AR of 0.28 ± 0.03 kgOC/m^2^/yr (0.63 ± 0.07 kgOM/m^2^/yr), that is 3x higher than that reported by Manies *et al*.^33^ for a coeval (<60 yr) layer of a boreal, moss-dominated rich fen, and an average AR of 3.75 ± 0.44 gTN/m^2^/yr, that is higher than that generally found in bogs, but in the range of AR of a variety of mires^33^. Quite similar values of AR were found also between 100 and 200 cm of depth, corresponding to a timescale spanning from *ca.*1919–1964 (Table 1). So that, 2 m of *Sphagnum* peat accumulated in ~100 years. Below 200 cm, the average AR of OM and OC strongly decreases (0.28 ± 0.07 and 0.14 ± 0.03 kg/m^2^/yr, respectively), whereas that of TN increases (6.54 ± 2.19 gTN/m^2^/yr), probably as a consequence of both decomposition and changes in the botanical composition of peat-forming plants. Then, higher AR of both elements (0.29 ± 0.22 kgOC/m^2^/yr and 9.91 ± 7.39 gTN/m^2^/yr) were recorded from 315 down to 400 cm of depth (AD 1292–1396 to AD 1492–1602) (Table 1). This results in estimated, average long-term (700 yr) AR of 0.17–0.19 kgOC/m^2^/yr and 5.6–6.6 gTN/m^2^/yr (Table 1). While long-term OC AR found here is 3–4x higher than that reported by Manies *et al*.^33^ for a 1,400 yr old boreal, moss-dominated rich fen (0.044 ± 0.005 kgC/m^2^/yr), long-term TN AR is 2x higher than the above mentioned fen (2.66 ± 0.14 gN/m^2^/yr; ref. 33), 6–7x higher than that reported for bogs (0.87 gN/m^2^/yr) by Wang *et al*.^19^ and 11–13x higher than that previously found for peatlands, in general (~0.5 gN/m^2^/yr; ref. 18). Moreover, while the decadal AR of OC was expected to be higher than the long-term one (1.4x), the decadal AR of TN was surprisingly lower (0.5x). The average long-term AR of bulk OM (0.34–0.40 kg/m^2^/yr; Table 1) is comparable to that reported by Zoltai^34^ for subarctic peatlands (0.332 kgOM/m^2^/yr) and ~35% lower than that reported for boreal peatlands (0.566 kgOM/m^2^/yr) during the <1,500 yr period. So, even in sub-Mediterranean climate conditions, characterized by two months summer drought, free-floating islands are important sinks of both elements, with AR comparable with, or higher than, other peatland ecosystems.

### How fast can *Sphagnum* peat accumulate?

^14^C age dating revealed that the peat column has accumulated in less than 700 yr (624 ± 21 yr BP; Cal AD 1293–1396) (Supplementary Table S4), resulting in an average growth rate (GR) of 0.52–0.61 cm/yr (Table 1): this value is, on average, 10x higher than normal in northern European bogs (0.02–0.08 cm/yr; ref. 35). As peat growth and increasing float thickness/weight might result in the separation of some peat material from the bottom of the island, the possibility that the island may have lost deeper older material cannot be excluded. What is surprising is that the top 110 cm, consisting of perfectly preserved *Sphagnum* material, is modern (130.9 ± 0.3 pMC) (Supplementary Table S4), and that, according to Goodsite *et al*.^36^, probably accumulated during the last ~50 yr (age also confirmed by ^210^Pb chronology; Supplementary Table S6). In fact, in the top 200 cm, the estimated average GR is *ca.* 2 cm/yr (Table 1). One peculiarity of this island is that the annual GR can be measured and not just estimated; in fact, within the top 20–30 cm, *Sphagnum* moss is separated by an annual input of leaves of *Populus tremula* (Supplementary Fig. S6). The average GR estimated at Posta Fibreno is extremely high (10x) compared with corresponding values from European continental peatlands, where the top 100 cm represents up to 2,200 yr of peat accumulation^35^,^37^,^38^, and GR averaging around 0.04–0.06 cm/yr.

The reason for such as abnormal GR in the top 200 cm may lie in the particular environment hosting the free-floating isle (Supplementary Information). *Sphagnum* mosses lack stomata, rhizoids and water conducting tissues; consequently, their growth, nutrition and vitality depend on the chemical composition of the surrounding water^39^. Generally speaking, since most *Sphagnum* species are sensitive to both high pH and increased concentrations of calcium (Ca) and bicarbonate (HCO_3^−^_) in pore water and surface water, their growth in floating systems is generally more complicated than in bogs^40^. Harpenslager *et al*.^22^, investigating the growth of four different *Sphagnum* species on peat floating on Ca- and HCO_3^−^_–rich water under laboratory conditions, found that most of them increased their initial biomass (by 600% in the case of *S. palustre*) and proportionally lowered the pH during a 12-weeks experiment. This is probably what occurred in the unbuffered and acidic first meter of the Posta Fibreno profile. Here, the high GR combined with low decomposition rate resulted in a fast build-up of the peat layer which slowly reduced the influence of the underlying calcareous water, thus creating an ombrotrophic environment. In fact, this free-floating island can be classified as a transitional mire with a secondary ombrotrophic local dominance induced by buoyancy. More details about the possible formation of the island and the ecology of *S. palustre* are reported in the Supplementary Information. The elevated concentration of dissolved CO_2_ occurring in the water of the karst lake (Supplementary Information) could also have played a positive effect on both photosynthetic and GRs, as hypothesized by several authors^41^,^42^.

### Implications for paleo-environmental reconstructions and geochemical studies

While cores from terrestrial peatlands, and ombrotrophic bogs in particular, have been often (and successfully) used to reconstruct climate and vegetation changes, as well as historical human activity during millennia^43^,^44^,^45^,^46^,^47^,^48^, almost no studies are present in literature about the use peat profiles from free-floating mires for such environmental studies. Reporting the pollen stratigraphy from a 300-cm deep floating mat located in a caldera on the Easter Island, Butler *et al*.^23^ found several anomalous radiocarbon ages throughout the profile; as the possibility that studied mat “flipped over” on more than one occasion could not be ruled out, they questioned the reliability of floating mats as chronological indicators or archives of vegetation changes.

At Posta Fibreno, this phenomenon was not observed; on the contrary, having this island a very high GR (0.5–0.6 cm/yr) compared to terrestrial peatlands (0.02–0.08 cm/yr), it shows a great potential to be used as archive of environmental changes. At the best of our knowledge, such a high detail has been never obtained in coeval peat archives.

**Figure 5:**
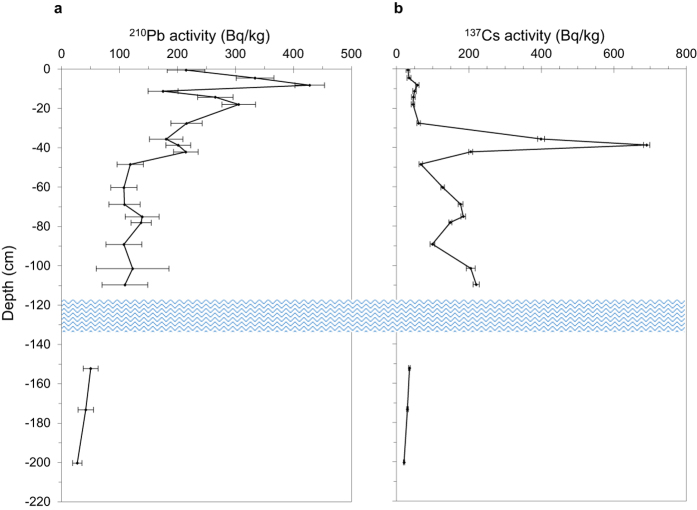
Concentrations of fallout radionuclides with depth. The figure illustrates the concentration of total ^210^Pb (**a**) and radiocaesium (**b**) throughout the top, 200 cm deep, section of the peat profile. The blue wavy line represents the water lens occurring throughout the profile. Supported ^210^Pb (*i.e.*, ^226^Ra) concentrations are extremely low (typically <2 Bq/kg), reflecting the highly OM content of the peat. Consequently, unsupported (fallout) ^210^Pb concentrations are scarcely distinguishable from total ^210^Pb concentrations. More details are reported in Supplementary Table S5.

Moreover, ^210^Pb and ^137^Cs activity recorded in selected samples throughout the profile (Fig. 5; Supplementary Table S5) shows a coherent trend, confirming the reliability of this possible record. Although ^210^Pb activity varied sometimes irregularly with depth, the general trend was a relatively steady decline in activity, from a maximum of 428 ± 26 Bq/kg in the near-surface samples to around 25 Bq/kg in the deepest sample analyzed (Fig. 5a; Supplementary Table S5), suggesting that the uppermost 200 cm of the core spans a period of around four ^210^Pb half-lives (~90–100 yr) (Supplementary Table S6). This is in agreement with ^14^C measurement carried out on samples PF2#97 and PFB1#09 (Supplementary Table S4) and is supported by calculations of the mean AR (0.07 g/cm^2^/yr or 2.2 cm/yr) determined from the mean gradient of the ^210^Pb profile^49^ (Supplementary Table S6). The ^137^Cs record (Fig. 5b) shows three distinct peaks (around 39, 75 and 110 cm of depth). The most prominent of these, at *ca.* 39 cm, records the fallout from the 1986 Chernobyl accident, whereas the two deeper peaks, at *ca.* 75 and 110 cm, may both date from the period of maximum fallout from the atmospheric testing of nuclear weapons in the early to mid-1960s (Supplementary Table S6 and Fig. S7). The separation of the peaks suggests a period of more rapid peat accumulation at that time. In support of this, it may be noted that ^210^Pb concentrations are relatively uniform over this part of the record. Further, the ^14^C results suggest near synchronous dates for samples PF2#97 and PFB1#09. More details are reported in Supplementary Information.

As most of the suitable natural archives, including peat bogs and glacial ices, are restricted to the alpine zone, and considering that the free-floating mire of Posta Fibreno probably consists of the southernmost European population of *Sphagnum*^25^, *i.e.*, one of the southernmost potential peat archives of Europe, it promises to provide new, high resolution reconstructions of atmospheric deposition and environmental change.

## Methods

A complete, 400-cm deep peat profile was collected on 18^th^ July 2012 from the central domed area of the Posta Fibreno free-floating island (41°41′41.8″ N; 13°41′30.3″ E; 290 m a.s.l.), where the surface peat layers are clearly elevated up to ~1 m beyond the edge of the isle. The uppermost 100-cm core (core PF2) was collected using a Wardenaar sampler; constructed entirely using a Ti-Al-Mn alloy, it allows removing a monolith 15 × 15 × 100 cm and minimizes compression. The remaining 300-cm were collected using a stainless steel Belarus corer, providing semi-cylindrical peat sections which are 50 cm long and 10 cm wide (Supplementary Fig. S1). Belarus cores (from PFB1 to PFB6) were collected in staggered intervals from two adjacent holes to provide a complete vertical peat profile, while avoiding the depths affected by the nose cone of the corer itself^50^. Once collected, Wardenaar and Belarus cores were photographed, wrapped in polyethylene cling film, placed in specifically-built boxes, brought to the lab and frozen at −18 °C. Wardenaar and Belarus cores were then cut while frozen in 1.0 ± 0.1 cm and 2.0 ± 0.2 cm slices, respectively, using a stainless steel band saw in the ultra-clean SWAMP lab (University of Alberta, Edmonton, Canada). The edges (*~*1 cm) were trimmed away from each slice.

Plant macrofossils were isolated from 1.5-cm^3^ subsamples removed from each even slice (*~*2 cm intervals). Four-to-five subsamples were then merged to obtain a significant amount of material to be analyzed; as a consequence, obtained final samples were representative, on average, of 11 ± 4 cm thick layers. Subsamples were dispersed in cold water; when the complete dispersion was achieved, samples were washed over 300 and 180-μm sieves to retain the larger fraction^51^. The identification and estimation of the abundance (volume percentage or absolute numbers) of peat-forming vegetation and charcoal particles was performed in Petri dishes (63.5 cm^2^) using a low-power Nikon microscope at 10–20x magnification. All identifiable and uncharred plant remains were picked out with a delicate brush and placed in another Petri dish containing a solution of distilled water, glycerol and 2% formalin (1:1:1) (ref. 52). In identifying macrofossils, the modern vegetation present on the island and in the area was taken into account.

Twelve samples were selected throughout the whole profile, prepared and analyzed for radiocarbon at the Beta Analytic Inc. laboratory (Miami, USA). Details about specimens analyzed for ^14^C dating by accelerator mass spectrometry (AMS) and pre-treatments carried out are reported in Supplementary Table S4. Generally speaking, in selecting material for AMS radiocarbon dating, *Sphagnum* remains (stems, branches and leaves) were preferred. For some samples, other above ground plant remains (stems, leaves) or silty peat were used to attain the minimum mass required for dating. The resulting ^14^C ages were calibrated using IntCal13 calibration curves^53^ and the clam software package^54^.

In addition to ^14^C, 21 dried samples were analyzed for ^210^Pb, ^226^Ra and ^137^Cs by direct gamma assay in the Liverpool University Environmental Radioactivity Laboratory using an Ortec HPGe GMX series coaxial low background intrinsic germanium detector^55^. ^210^Pb was determined via its gamma emissions at 46.5 keV, ^226^Ra by the 295 keV and 352 keV γ-rays emitted by its daughter isotope ^214^Pb, and ^137^Cs by its emissions at 662 keV. The spectra were also examined for the 59.5 keV ^241^Am photo-peak but, in all samples analyzed, emissions from this radionuclide were found to be below the minimum level of detection (~0.002 Bq). The absolute efficiencies of the detectors were determined using calibrated sources and sediment samples of known activity. Corrections were made for the effect of self-absorption of low energy γ-rays within the sample^56^. ^210^Pb dates then were calculated using the Constant Rate of Supply (CRS) model^57^.

Bulk density was calculated by dividing the volume of each peat sample by the corresponding dry weight, determined drying samples at 105 °C for 24 hr. The ash content, expressed as a percentage of the initial dry weight, was determined on the outside edges of each peat sample by combustion in a muffle furnace at 550 °C for 12 hr. The water content was determined using equations (1) and (2):


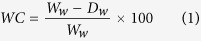


where *WC* is the water content (in %), while *W*_*w*_ and *D*_*w*_ are the wet and the dry weight (at 105 °C), respectively;


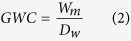


where *GWC* is the gravimetric water content (as g_water_/g_peat_), *W*_*m*_ is the mass of water and *D*_*w*_ is the weight of dry peat.

pH and EC were determined on unfiltered porewater samples collected using the squeezing technique proposed by Shotyk and Steinmann^58^; a Philips pH meter equipped with a Hanna Instruments HI 1230 probe was used for the pH determination, whereas EC was measured through a XS Cond 510 conductivity meter.

Total C, H, N and S concentrations in peat samples were determined by dry combustion using an elemental analyzer (Fisons EA1108, Milan, Italy). The instrument was calibrated using both BBOT [2,5-Bis-(5-tert-butylbenzoxazol-2-yl)-thiophen] and Phenanthrene Enriched standard (Fison/Carlo Erba Italia s.p.a.). Total organic C was determined by difference between total C and inorganic C (TOC = TC–IC), the latter one determined, using the same elemental analyzer, on peat samples pre-dried at 420 °C for 12 h. In this case, urea was used as standard. Oxygen content was calculated by difference: O% = 100 - (C + H + N + S)%. All samples were analyzed in triplicate, and obtained data corrected for ash and moisture content. Ratios between elements (*i.e.*, C/N, H/C and O/C) have been determined as atomic ratios, considering as C concentration those of the TOC. Total C and N mass per unit area has been calculated as the product of their concentration (mg/g), bulk density (g/cm^3^) and thickness of each slice (cm); then, their content has been integrated over the depth of the core, and expressed as mass per unit area of the bog surface (kg/m^2^).

Isotopic ratios ^13^C/^12^C and ^15^N/^14^N were determined on selected peat samples (*n* = 45) by Continuous Flow-Isotope Ratio Mass Spectrometry (CF-IRMS, Delta Plus, ThermoFisher) coupled with an elemental analyser (CHNS-O mod. EA 1110 ThermoFisher). The Elemental Analyser was interfaced with the IRMS through the Conflo III (Thermo) dosing the samples and the reference gasses (N_2_ and CO_2_). The pyrolyser was equipped with a PorapackQS (GC column). Isotopic values were expressed in δ(‰) relative to V-PDB (Vienna-Pee Dee Belemnite) for carbon (δ^13^C), and AIR (Atmospheric nitrogen) for nitrogen (δ^15^N), according to equation (3):


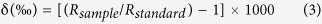


where *R*_sample_ and *R*_standard_ are the ^13^C/^12^C or ^15^N/^14^N ratios of sample and standard, respectively. All analyses were carried out in triplicate. For δ^13^C determination, peat samples were subjected to acid fumigation^59^ before the analysis to exclude the influence of carbonates.

The FT-IR spectra were acquired on selected peat samples (*n* = 60) in transmittance mode using a Thermo Nicolet Nexus FTIR Spectrophotometer equipped with Nicolet Omnic 6.0 software. Potassium bromide pellets were obtained by pressing, under vacuum, a homogenized mixture of 400 mg of infrared grade KBr and 1 mg of dry sample. Spectra were recorded in the range 4000–400 cm^−1^, with a 2 cm^−1^ resolution and with 64 scans for each acquisition.

Elemental analysis, CF-IRMS and FT-IR were carried out on dried and finely milled peat material; grinding was done using a Retsch agate ball mill (PM 400, Haan, Germany) with 250 mL jars containing three 30 mm balls at 300 rpm for four × 2.3 minutes (first forward, then reverse, then forward, then reverse).

Statistical correlations were performed using the Statistica Version 7 software (StatSoft Inc., 2004). Significant differences were calculated on the basis of the Tukey’s test, considering a significance level of *p* < 0.05.

## Additional Information

**How to cite this article**: Zaccone, C. *et al*. Highly anomalous accumulation rates of C and N recorded by a relic, free-floating peatland in Central Italy. *Sci. Rep.* **7**, 43040; doi: 10.1038/srep43040 (2017).

**Publisher's note:** Springer Nature remains neutral with regard to jurisdictional claims in published maps and institutional affiliations.

## Supplementary Material

Supplementary Information

## Figures and Tables

**Table 1:** Estimated average accumulation rates of peat organic matter (OM), organic C (OC) and total N (TN) at both long-term (0–400 cm; *ca.* 700 yr) and short-term (from 1964 ± 4 to 2012, *i.e.* 0–101 cm; from 1919 ± 10 to 1964 ± 4, *i.e.* 101–200 cm) timescales. Corresponding, estimated average growth rates (GR) are also reported*.

	0–400 cm			0–101 cm			101–200 cm			200–316 cm			316–400 cm		
	min	max	avg ± SD	min	max	avg ± SD	min	max	avg ± SD	min	max	avg ± SD	min	max	avg ± SD
OM (kg/m^2^/yr)	0.34	0.40	0.37 ± 0.04	0.58	0.68	0.63 ± 0.07	0.52	0.99	0.76 ± 0.33	0.23	0.33	0.28 ± 0.07	0.27	0.87	0.57 ± 0.42
OC (kg/m^2^/yr)	0.17	0.19	0.18 ± 0.02	0.25	0.30	0.28 ± 0.03	0.24	0.46	0.35 ± 0.15	0.11	0.16	0.14 ± 0.03	0.14	0.44	0.29 ± 0.22
TN (g/m^2^/yr)	5.63	6.58	6.10 ± 0.67	3.44	4.06	3.75 ± 0.44	4.02	7.66	5.84 ± 2.57	4.99	8.08	6.54 ± 2.19	4.69	15.14	9.91 ± 7.39
GR (cm/yr)	0.52	0.61	0.56 ± 0.06	2.07	2.44	2.25 ± 0.27	1.37	2.61	1.99 ± 0.88	0.26	0.37	0.31 ± 0.08	0.24	0.77	0.50 ± 0.37

^*^The following age dates have been used for the estimation of minimum and maximum values of accumulation and growth rates: AD 1964 ± 4 for PF B1#04 (at *ca.* 101 cm), AD 1919 ± 10 for PF B3#03 (at *ca.* 200 cm), AD 1492–1602 for PF B5#11 (at *ca.* 316 cm), and AD 1292–1396 for PF B6#19 (at *ca.* 385 cm) (see Supplementary Tables S3 and S6 for details about radiocarbon and ^210^Pb age dates, respectively).
