# Supplementary material for: Highly anomalous accumulation rates of C and N recorded by a relic, free-floating peatland in Central Italy
Source: Sci Rep. 2017 Feb 23;7:43040. doi: 10.1038/srep43040 (PMC5322321; doi:10.1038/srep43040)
Supplement: Supplementary Information [file srep43040-s1.pdf]

Highly anomalous accumulation rates of C and N recorded by a relic, free-floating peatland in Central Italy

Claudio Zaccone*, Daniela Lobianco, William Shotyk, Claudio Ciavatta, Peter G. Appleby, Elisabetta Brugiapaglia, Laura Casella, Teodoro M. Miano, Valeria D'Orazio

Supplementary Information

(22 pages, 7 figures, 6 tables)

1. **The Protected Area "Lake of Posta Fibreno"**

    1.1. The free-floating island: vegetation patterns

2. **Peat core collection**
3. **Botanical composition of peat**

    3.1. Ecology of Sphagnum palustre

4. **Main physical and chemical properties of the 4m-deep peat core**
5. **Organic matter evolution throughout the 4m-deep peat profile**
6. **Radiocarbon age dating**
7. **Evidence of annual accumulation of *Sphagnum* moss**
8. **Concentration of fallout radionuclides and ^{210}Pb core chronology**
9. **References**

* Corresponding author
E-mail address: claudio.zaccone@unifg.it; *URL:* http://www.claudiozaccone.net

1. The Protected Area "Lake of Posta Fibreno"

The protected area of "Lake of Posta Fibreno" (code: SIC-ZPS IT6050015) is located in the Lazio region, Central Italy, and was established in 1983. It has a surface of *ca.* 139 ha, 55% of which consist of the homonymous lake having the Fibreno river as exclusive inflow (Habitat code N06), 27% of bogs, marshes, water fringed vegetation and fens (Habitat code N07), 10% of heath, scrub, maquis, garrigue and phygrana (Habitat code N08), 5% of humid and mesophile grassland (Habitat code N10), 2% of arable land (Habitat code N15) and 1% of other land (Habitat code N23).

The Posta Fibreno lake (290 m a.s.l.) is a spring lake finding its recharge area in the karstic environment of carbonate Apennines[60]. The lake takes its origin from a complex of submerged and surface karstic springs of mineral-rich water[24]. The waters of the lake have a neutral pH value and a pCO_2 ranging from 0.010 to 0.046 bar; main ions present are HCO_3^-, Ca^{2+} and Mg^{2+} due to the dissolution of the calcareous rocks by the circulating water[61]. The average, annual temperature of the lake is *ca.* 10-11°C, whereas the depth is around 2.7 m, except in the submerged dolines, where it may reach a depth of *ca.* 15 m (ref. 61). One of this submerged doline (sinkhole), annexed to the easternmost edge of the lake and having a depth of *ca.* 10-11 m, hosts the free-floating island of "La Rota" (0.07 ha). Here, annual mean values reported by Mastrantuono et al.[62] for the lake water (at site 3) are the following: pH = 7.14, EC = 601 μS/cm, TDS = 343 mg/l, T = 10.4°C, O_2 = 10.6 mg/l, NO_3^- = 2.2 mg/l, PO_4^{3-} = 0.08 mg/l, HCO_3^- = 402 mg/l.

Deep piping sinkholes in the Latium region are well-known and documented natural phenomena[63,64]. Their formation is a quite common phenomenon also in the Fibreno catchment because of the presence of thick carbonate deposits that are susceptible to dissolution due to circulating groundwater[24]. Here, bubbling phenomena caused by the high concentrations of free and dissolved gases (mainly CO_2, together with CH_4, H_2S) can be easily and frequently observed. The occurrence of these gases could both have a deep-origin[63] and be caused by the decomposition of the organic matter (OM) accumulated at the bottom of the lake.

Geological evidence point out the existence in the area of a large lacustrine basin since Late Pleistocene. The progressive filling of the lake, due to changing in climatic conditions and neotectonic events, caused the formation of peat deposits in the area, following different depositional cycles in a swampy environment[24,65].

1.1. The free-floating island: vegetation patterns

The free-floating island of Posta Fibreno is colonized by both herbaceous and phanerophytic plant species, presenting a concentric sorting of belts of vegetation dominated, starting from the outer belt, by *Carex paniculata*, *Salix cinerea* and *Populus tremula*, with a core of *Sphagnum palustre* hummocks. Behind the geobiological formation, the entire local floristic species-pool hosted on the island is of extremely high importance for conservation, representing a nucleus of representatives from Central Europe vegetation (e.g., *Peucedanum palustre*, *Carex elata*, *Caltha palustris*) in a Mediterranean scenario (olive groves are common in the surrounding hills where the native flora is dominated by *Quercus pubescens* and broadleaved evergreen sclerophyllous shrubs).

Here, *S. palustre*, that occupies the island's inner core beneath a cover of *P. tremula* trees up to 15 m high, is the most fragile and striking element of the island itself. More details about the present vegetation on the free-floating isle are reported elsewhere[24,66].

2. Peat core collection

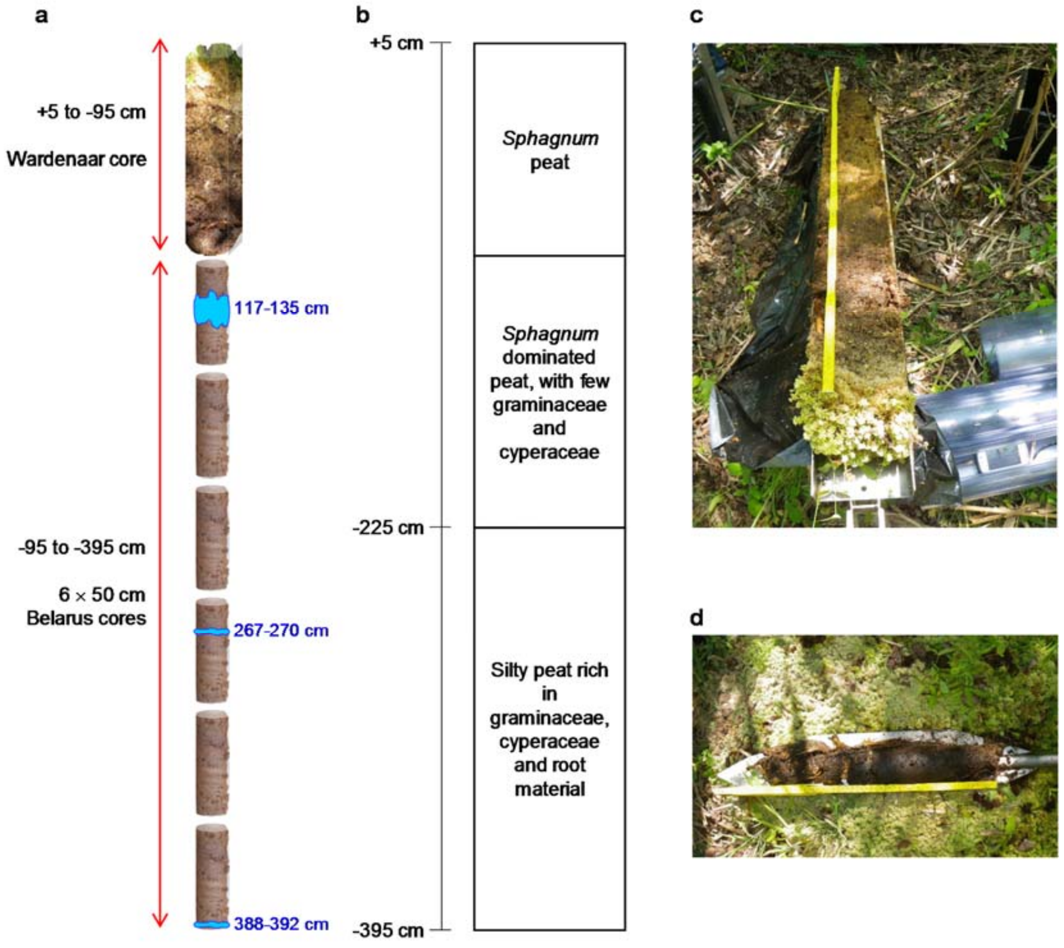

Supplementary Figure S1. Schematic representation of the peat core collected at Posta Fibreno.

(a) Peat core collection; the top 100 cm monolith (PF2) was collected using a Wardenaar corer[67], whereas the bottom 300 cm were collected using a Belarus corer[68] (6 cores, 50 cm each; from PFB1 to PFB6). Blue layers represent water lenses occurring throughout the profile. **(b)** Schematic representation of macrofossil stratigraphy throughout the 400 cm profile. **(c)** The 100-cm peat monolith (PF2) collected using the Wardenaar corer, consisting almost exclusively of *Sphagnum palustre* (photo by C.Z.). **(d)** One of the collected Belarus cores (*i.e.*, PFB5, from 300 to 350 cm of depth) (photo by C.Z.).

3. Botanical composition of peat

The free-floating island of Posta Fibreno can be classified as a transitional mire with a secondary ombrotrophic local dominance induced by buoyancy. The increase in the abundance of *Graminaceae* and *Cyperaceae*, as well as of roots and wood fragments, below 200 cm of depth (Supplementary Fig. S2) proves that the original peat accumulation occurred in a fen environment. *Sphagnum* was probably already in the local plant community stock also if with a low importance in coverage, being controlled in its distribution by the minerotrophic conditions induced by the oscillation of water level before the buoyancy occurred. Therefore, the increment of *Sphagnum* probably witnesses the moment of hydrogeological isolation of the inner-core of the free-floating island, *i.e.*, when precipitations become the only source of water and nutrients Further details about the vegetation history at Posta Fibreno are reported elsewhere[69].

3.1. Ecology of Sphagnum palustre

Sphagnum palustre, a species of the Eurosibiric geoelement, is a widespread moss found in habitats that are neither highly calcareous nor highly acidic[70].

The remote reason of its occurrence in the original wetlands of Fibreno Lake is to be related to its minerotrophic requirements[71]. The species is one of the more shade-tolerant, commonly present in carr (black alder woods), but in more open situations it occurs along mire margins or even in sunny wetlands[72,73]. In Italy, this species exhibits scattered and fragmentary populations all along the Apennine chain, revealing a long-going process of range reduction due to desiccation since Middle Holocene[74] and human destruction of wetland habitats. Studies on long-term dynamics of *Sphagnum* established that a clone of *Sphagnum* can persist within the plot for centuries[75]. As old respiring tissues are gradually buried in peat, the growing capitula are eternally young and the shoots we see on the mire surface today may well be the same genetic individuals that was established when the mire was formed hundreds of years ago.

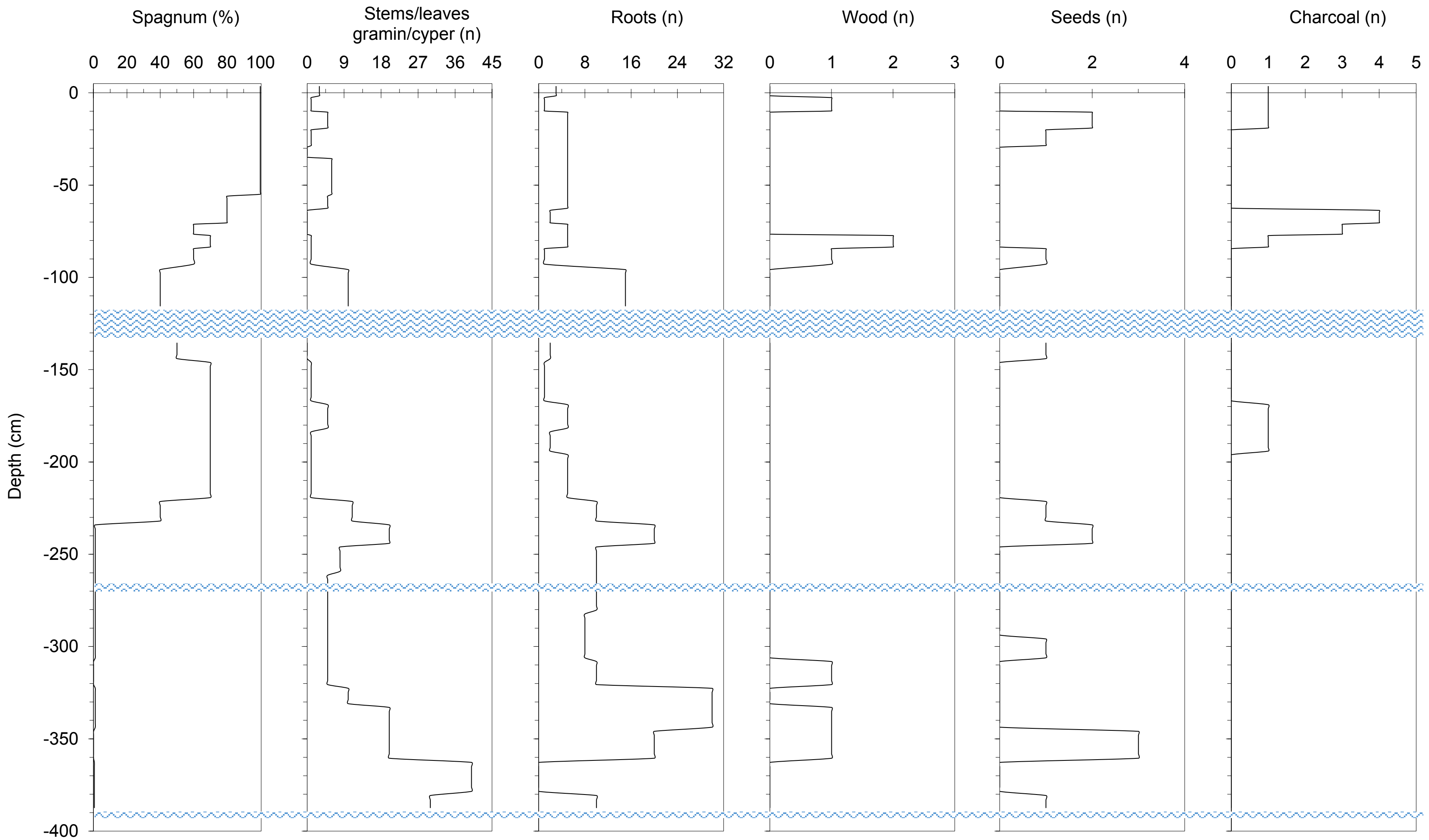

Supplementary Figure S2. Plant macrofossils diagram for the free-floating island of Posta Fibreno.

4. Main physical and chemical properties of the 4m-deep peat core

The first 220 cm show a very low bulk density (avg., 0.035±0.012 g/cm^3), an average ash content <6% and a pH generally increasing with depth (from 4.1 at the surface to 7.4 at 112 cm of depth, and then constantly around 7.1±0.1 down to 220 cm) (Fig. 2; Supplementary Table S1 and Fig. S3). In particular, in the top 100 cm, *i.e.*, the section emerging above the lake surface, the *Sphagnum* material is so well preserved that it is hard to classify it as "peat"[76].

The bottom 180 cm show very different properties, including greater bulk density (avg., 0.117±0.044 g/cm^3) and ash content (avg., 9.3±5.3%) that reach their maximum between 300-325 cm (0.27 g/cm^3 and 22%, respectively) (Fig. 2; Supplementary Table S1). From 220 to 400 cm of depth, the pH is rather constant and averages around 7.0±0.1 (Supplementary Table S1 and Fig. S3), because of the acid buffering caused by the bicarbonate-rich waters[41] characterizing this karst lake.

The gravimetric water content (GWC; Fig. 2) mirrors very well the different botanic composition and degree of decomposition of the peat throughout the profile; in fact, it averages around 26.6 g_{water}/g_{peat} in the top 220 cm, and around 11.4 g_{water}/g_{peat} in the remaining 180 cm of depth (Supplementary Table S1), and shows a significant correlation with both density (negative) and main atomic ratios (positive) (Supplementary Table S2). A similar trend is observed for water content (WC) (Fig. 2; Supplementary Table S2).

Supplementary Table S1. Maximum, minimum, average (± st. dev.) and median values of main physical and chemical properties of the peat at Posta Fibreno.

	**Ash** (%)	**Dry Density** (g/cm^3)	**Wet density** (g/cm^3)	**WC** (%)	**GWC** (g_{water}/g_{peat})	**pH**	**EC** (µS/cm)	**C** (%)	**N** (%)	**C/N**	**H/C**	**O/C**	**δ^{13}C** (‰)	**δ^{15}N** (‰)
Whole profile														
max	22.5	0.272	1.87	98.0	47.9	7.4	1006	50.5	2.95	130.7	1.95	1.19	-25.77	4.01
min	0.8	0.017	0.37	81.3	4.3	4.1	50	35.1	0.34	16.9	1.27	0.54	-30.22	-3.41
average	6.9	0.063	1.04	94.4	21.5	6.0	154	43.0	1.00	67.5	1.56	0.77	-27.25	-0.39
st. dev.	3.7	0.047	0.29	3.1	9.6	1.2	121	3.7	0.63	30.1	0.17	0.15	1.03	1.84
median	6.1	0.043	0.99	95.3	20.4	6.6	122	43.3	0.70	70.0	1.56	0.76	-27.01	-0.90
Top 220 cm														
max	10.6	0.079	1.58	98.0	47.9	7.4	1006.0	46.9	1.11	130.7	1.95	1.19	-25.77	-0.31
min	0.8	0.017	0.37	93.5	14.3	4.1	64.0	35.1	0.34	44.9	1.46	0.65	-30.22	-3.41
average	5.8	0.035	0.91	96.1	26.6	5.7	158.2	41.6	0.61	85.2	1.65	0.84	-27.42	-1.68
st. dev.	1.7	0.012	0.22	1.0	7.3	1.2	126.1	3.2	0.17	19.7	0.13	0.13	1.26	0.77
median	5.6	0.032	0.87	96.3	25.8	5.5	123.0	42.6	0.57	85.3	1.60	0.79	-26.96	-1.66
Bottom 180 cm														
max	22.5	0.272	1.87	95.5	21.1	7.3	180.0	50.5	2.95	51.0	1.60	0.80	-25.89	4.01
min	3.2	0.051	0.98	81.3	4.3	6.9	50.0	35.4	0.97	16.9	1.27	0.54	-27.74	-0.62
average	9.3	0.117	1.31	91.1	11.4	7.0	117.4	45.6	1.75	32.6	1.39	0.63	-27.02	1.48
st. dev.	5.3	0.044	0.21	3.0	3.6	0.1	53.6	3.3	0.47	9.8	0.06	0.06	0.46	1.24
median	7.1	0.112	1.32	91.4	10.6	7.0	120.0	46.4	1.65	32.2	1.37	0.63	-27.09	1.24

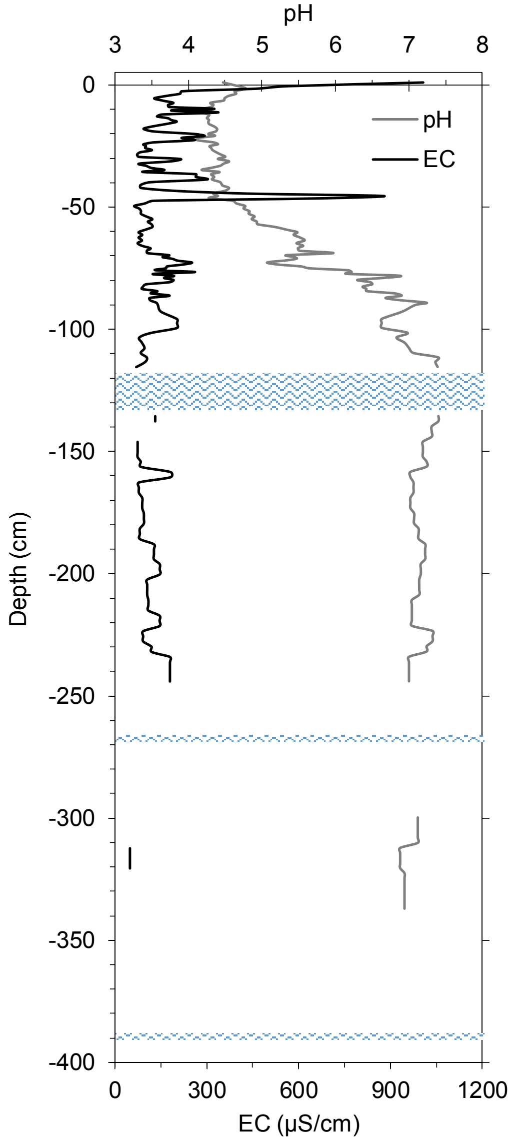

Supplementary Figure S3. pH and EC variation throughout the profile. Blue wavy lines represent water lenses occurring throughout the profile.

In some samples, the extracted porewater volume was not sufficient enough to allow these two parameters to me measured. The increase of pH recorded throughout the Posta Fibreno floating island profile could be a consequence of the acid buffering by bicarbonate. An increase in pH to circumneutral values (due to acid buffering by HCO_3^-), which occurs in deeper peat layers, accelerates CH_4 production due to the increased availability of H_2+CO_2 or acetate caused by the stimulation of microbial hydrolysis of complex polymers or by a direct effect of increased pH on methanogenic Archaea[41].

Supplementary Table S2. Linear correlation coefficients among main physical and chemical parameters. For all parameters, n = 229, with the exception of stable isotopes (n = 45). Significant (p<0.05) correlations are shown in red.

	**Density**	**WC**	**GWC**	**Ash**	**C/N**	**H/C**	**O/C**	**$\delta^{13}C$**	**$\delta^{15}N$**
**Density**	1.000<br>p = ---								
**WC**	-0.9492<br>p = 0.000	1.000<br>p = ---							
**GWC**	-0.7957<br>p = 0.000	0.8609<br>p = 0.000	1.000<br>p = ---						
**Ash**	0.6806<br>p = 0.000	-0.6254<br>p = 0.000	-0.3947<br>p = 0.000	1.000<br>p = ---					
**C/N**	-0.7711<br>p = 0.000	0.7337<br>p = 0.000	0.7579<br>p = 0.000	-0.5512<br>p = 0.000	1.000<br>p = ---				
**H/C**	-0.6946<br>p = 0.000	0.6727<br>p = 0.000	0.7830<br>p = 0.000	-0.3667<br>p = 0.000	0.7670<br>p = 0.000	1.000<br>p = ---			
**O/C**	-0.6179<br>p = 0.000	0.6310<br>p = 0.000	0.7739<br>p = 0.000	-0.3092<br>p = 0.001	0.6937<br>p = 0.000	0.9370<br>p = 0.000	1.000<br>p = ---		
**$\delta^{13}C$**	0.1513<br>p = 0.327	0.0317<br>p = 0.838	0.2232<br>p = 0.145	0.5077<br>p = 0.010	-0.1536<br>p = 0.319	-0.1620<br>p = 0.293	-0.1887<br>p = 0.220	1.000<br>p = ---	
**$\delta^{15}N$**	0.8172<br>p = 0.000	-0.7456<br>p = 0.000	-0.6701<br>p = 0.000	0.4534<br>p = 0.023	-0.8628<br>p = 0.000	-0.7814<br>p = 0.000	-0.7455<br>p = 0.000	0.2746<br>p = 0.071	1.000<br>p = ---

5. Organic matter evolution throughout the 4m-deep peat profile

Atomic ratios have been often used to describe decomposition processes throughout peat profiles[29,30,77,78]. Here, C/N, H/C and O/C ratios, that are positively correlated among each other (p<0.001) and negatively with density and $\delta^{15}N$ (p<0.001) (Supplementary Table S2 and Fig. S4), generally decrease with depth, suggesting the occurrence of more humified materials below 220 cm of depth (Fig. 4). In fact, it is assumed that the increase in peat humification is associated with a decrease in C/N, H/C and O/C ratios (as a relative enrichment of N relative to C, as well as a residual enrichment of more recalcitrant aromatic and aliphatic compounds, occur), with a ^{15}N enrichment and with an increase of density.

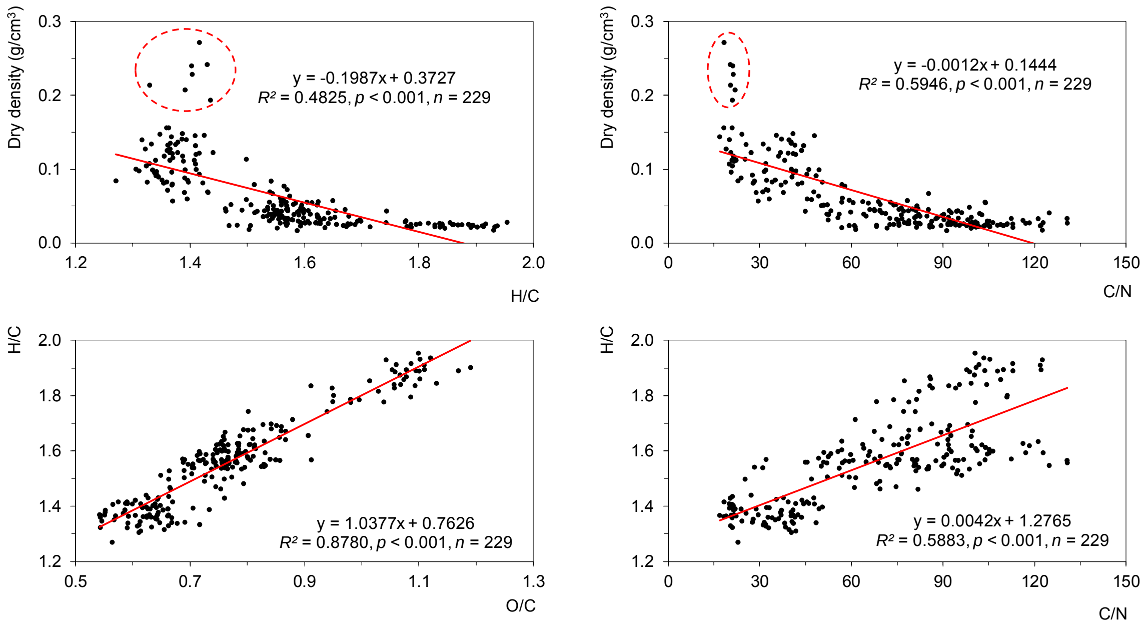

Supplementary Figure S4. Linear regressions among density and atomic ratios. Dotted circles surround samples between 300 and 320 cm of depth, whose high density values were mainly due to the high ash content rather than to more decomposed material. Details about statistical correlation among all parameters investigated are reported in Supplementary Table S2.

Observed trends are also in agreement with the WC and GWC, both positively correlated with all atomic ratios ($p<0.001$) (Supplementary Table S2); in fact, less decomposed peat generally retains more water than humified peat. Besides the humification degree, variation in density, WC, GWC, $\delta^{15}N$ and atomic ratios mirror also differences of peat-forming plants, which the dominance of *Sphagnum* in the top 220 cm, and reed-fen peat and silty peat rich in reeds down to 400 cm of depth (Supplementary Fig. S1 and S2).

The occurrence of very well preserved (almost undecomposed) *Sphagnum* material in the top 100 cm, and a more humified OM below *ca.* 200 cm of depth, is very clear also from FT-IR spectra (Supplementary Fig. S5). In fact, spectra recorded on samples selected from the first 100 cm are characterized by a number of absorption bands, with various relative intensities, ascribable to the presence of cellulosic, hemi-cellulosic, ligno-cellulosic, lignin-derived structures and other plant by-products that typically occur in the top, few centimeter-deep layers of *Sphagnum*-dominated peatlands[29,30,79]. Below 200 cm of depth, FT-IR spectra show the same main absorption bands, although differences in their relative intensity are evident, together with additional absorption peaks (Supplementary Table S4), suggesting that OM undergone a more pronounced decomposition process.

In particular, it is possible to observe with depth: *i)* an increase in the aromatic/aliphatic ratio, as underlined by a lower absorption intensity at *ca.* 2920–2850 cm^{-1} (aliphatic C-H stretching) and a simultaneous relative increase in absorption intensity at 1615–1625 cm^{-1} (mainly aromatic C=C stretching); *ii)* a general decrease until to the complete disappearance of the peak at about 1720 cm^{-1} (C=O stretching of carbonyl and carboxyl groups); *iii)* a light increase of the sharp peak at about 1510-1515 cm^{-1} (N-H and C-N associated bending of secondary amides, amide II band); *iv)* an increase in the relative intensity of the peaks at 1410 cm^{-1} (O-H deformation and C-O stretching of phenolic groups), 1260 cm^{-1} (C–O–C asymmetric stretching of aryl ethers) and 1270 cm^{-1} (-C-OH stretching of phenolic OH); and *v)* the disappearance of the peaks at 1063 cm^{-1}, ascribed to C-O stretching of aliphatic primary

alcohols and the simultaneous appearance of an overlapping band, characterized by two distinct peaks at about 1070 cm^{-1} and 1034 cm^{-1} (respectively, mineral impurities and/or S functional groups, such as C=S, S=O, and stretching of polysaccharides or polysaccharide-like substances).

Supplementary Table S3. Major IR absorption bands observed in peat samples, and corresponding assignments.

Wavenumber (cm^{-1})	Assignment
3420	O-H stretching, N-H stretching (minor), hydrogen-bonded OH
2925-2850	Asymmetric and symmetric stretching of aliphatic C-H
1725-1710	C=O stretching of COOH, aldehydes and ketones
1660-1630	C=O stretching of amide groups (amide I band), C=O of quinones and/or H-bonded conjugated ketones
1620-1600	Aromatic C=C stretching , COO^- symmetric stretching
1510	N-H and C-N associated bending of secondary amides (amide II band)
1460-1440	C-H asymmetric bending of CH_3 groups
1420-1410	O-H deformation and C-O stretching of phenolic groups
1400-1380	COO^- antisymmetric stretching, C-H bending of CH_2 and CH_3 groups
1361-1359	C-N stretching of tertiary amines
1289	C-N stretching of aromatic secondary amides
1270	-C-OH stretching of phenolic OH
1260	C–O–C asymmetric stretching of aryl ethers
1240	C-O stretching and OH deformation of COOH
1150	C-O stretching of aliphatic tertiary alcohols
1100	C-O stretching of aliphatic secondary alcohols
1070	mineral impurities and/or stretching of sulfur functional groups, such as C=S, S=O
1060-1050	C-O stretching of aliphatic primary alcohols
1040-1020	C-O stretching of polysaccharides or polysaccharide-like substances
990-700	C-H out-of-plane bending of benzene ring differently substituted

The decrease of the oxidation degree occurring throughout the whole profile confirms the presence of a more aromatic OM with depth (especially below 220 cm), as also underlined by decreasing values of the H/C and O/C ratios (Fig. 4b,c). Peat samples from 220 cm down to 400 cm of depth are also characterized by a gradual, relative increase of N-containing functional groups, resulting in the observed decrease in the C/N ratio in this region of the profile (Fig. 4a).

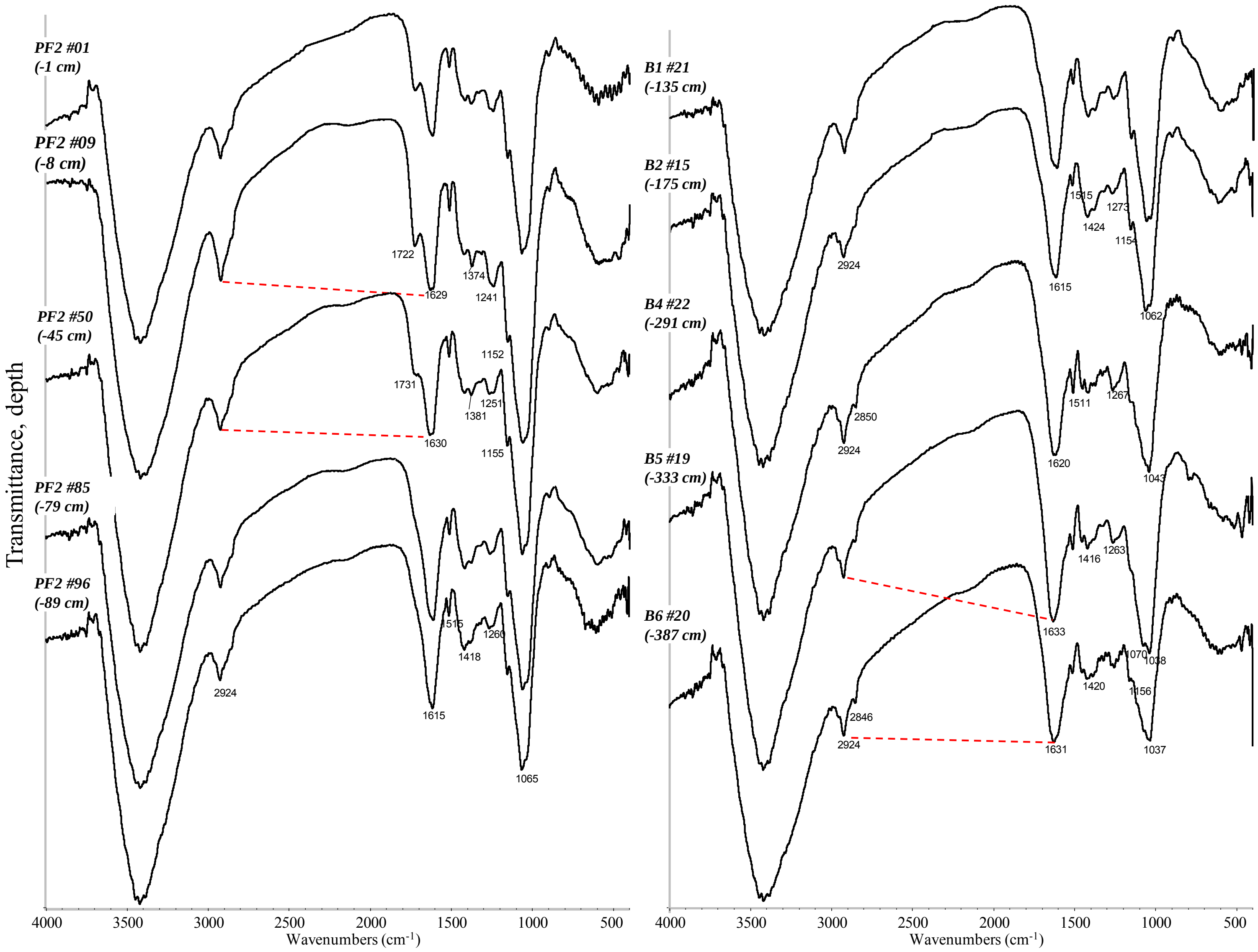

Supplementary Figure S5. FT-IR spectra of selected (n = 10) peat samples. Major absorption bands and corresponding assignment are reported in Supplementary Table S3.

6. Radiocarbon age dating

Supplementary Table S4. Radiocarbon age dates.

Sample code	Avg depth (cm)	(material): pre-treatment	Conventional age (^{14}C BP)	Percent modern C (pMC)	Fraction modern	$D^{14}C$ (‰)	Calibrated ages (2σ)	Interpolated ages [§]	Calibrated ages (2σ)
							Cal AD (95% probability)		Cal AD (68% probability)
PF2 #97	-90.1	(*Sphagnum*):<br>acid/alkali/acid	164.9±0.3 pMC	164.9±0.3 pMC	1.6494±0.0033	649.4±3.3			
PF B1#09	-111.5	(*Sphagnum*):<br>acid/alkali/acid	130.9±0.3 pMC	130.9±0.3 pMC	1.3085±0.0033	308.5±3.3			
PF B2#21 †	-187.9	(*Sphagnum*):<br>acid/alkali/acid	194±21 BP	97.6±0.3 pMC	0.9761±0.0026	-23.9±2.6	1657-1683<br>1735-1806<br>1930-Post 1950	1658-1683 (*21.3*)<br>1736-1805 (*53.1*)<br>1932-Post 1950 (*20.5*)	1665-1678<br>1765-1785<br>1794-1900<br>1940-Post 1950
PF B3#15	-221.4	(plant material):<br>acid/alkali/acid	298±18 BP	96.4±0.2 pMC	0.9636±0.0022	-36.4±0.2	1521-1591<br>1620-1647	1521-1592 (*67.7*)<br>1619-1648 (*27.1*)	1526-1555<br>1633-1644
PF B3#21 ‡	-238.3	(silty peat):<br>acid washes	332±21 BP	96.0±0.3 pMC	0.9595±0.0025	-40.5±2.5	1476-1642	1486-1604 (*75.4*)<br>1608-1639 (*19.4*)	1494-1528<br>1545-1601<br>1615-1634
PF B4#21	-289.0	(plant material):<br>acid/alkali/acid	278±22 BP	96.6±0.3 pMC	0.9660±0.0026	-34.0±2.6	1524-1557<br>1631-1659	1521-1579 (*45.9*)<br>1620-1663 (*47.0*)	1640-1649
PF B5#11	-316.4	(silty peat):<br>acid washes	315±23 BP	96.2±0.3 pMC	0.9615±0.0028	-38.5±2.8	1489-1646	1492-1602 (*74.4*)<br>1615-1644 (*20.5*)	1520-1593<br>1619-1642
PF B5#19	-333.0	(silty peat):<br>acid washes	411±21 BP	95.0±0.3 pMC	0.9501±0.0025	-49.9±2.5	1442-1484<br>1605-1606	1437-1492 (*90.7*)	1446-1464
PF B6#06	-356.1	(plant material):<br>acid/alkali/acid	435±21 BP	94.7±0.2 pMC	0.9473±0.0022	-52.7±2.2	1431-1470	1431-1470 (*95.0*)	-
PF B6#13	-371.7	(silty peat):<br>acid washes	519±21 BP	93.7±0.3 pMC	0.9374±0.0025	-62.6±2.5	1405-1435	1398-1439 (*95.0*)	1411-1427
PF B6#19	-385.3	(silty peat):<br>acid washes	624±21 BP	92.5±0.2 pMC	0.9253±0.0024	-74.7±2.4	1293-1328<br>1341-1396	1292-1328 (*37.5*)<br>1341-1396 (*57.4*)	1299-1319<br>1351-1370<br>1380-1390
Only for comparison:									
PF B6#19	-385.3	(plant material):<br>acid/alkali/acid	492±21 BP	94.1±0.3 pMC	0.9406±0.0025	-59.4±2.5	1413-1442	Not modelled	1420-1437

[§] For each interpolated age range, corresponding probability is reported in italic. Probability values <5% are not listed.

† The ^{210}Pb dates calculated using the CRM model date sample PF B2#21 between 1919±10 and 1938±7, *i.e.*, in the ^{14}C calibrated age range 1930-Post 1950.

‡ Sample PF B3#21 seems apparently and anomalously older compared to the two samples below (*i.e.*, PF B4#21 and PF B5#11). This is probably due to the high range width of their corresponding interpolated cal. ages (>130 yr). In fact, although the possibility of "flipping over" of these free-floating mats was not excluded by Butler et al.[23], it seems unlikely for the studied island based on plant macrofossil evidence. As a consequence, the ^{14}C calibrated age range 1608-1639 seems to be the more possible one for this sample.

7. Evidence of annual accumulation of *Sphagnum* moss

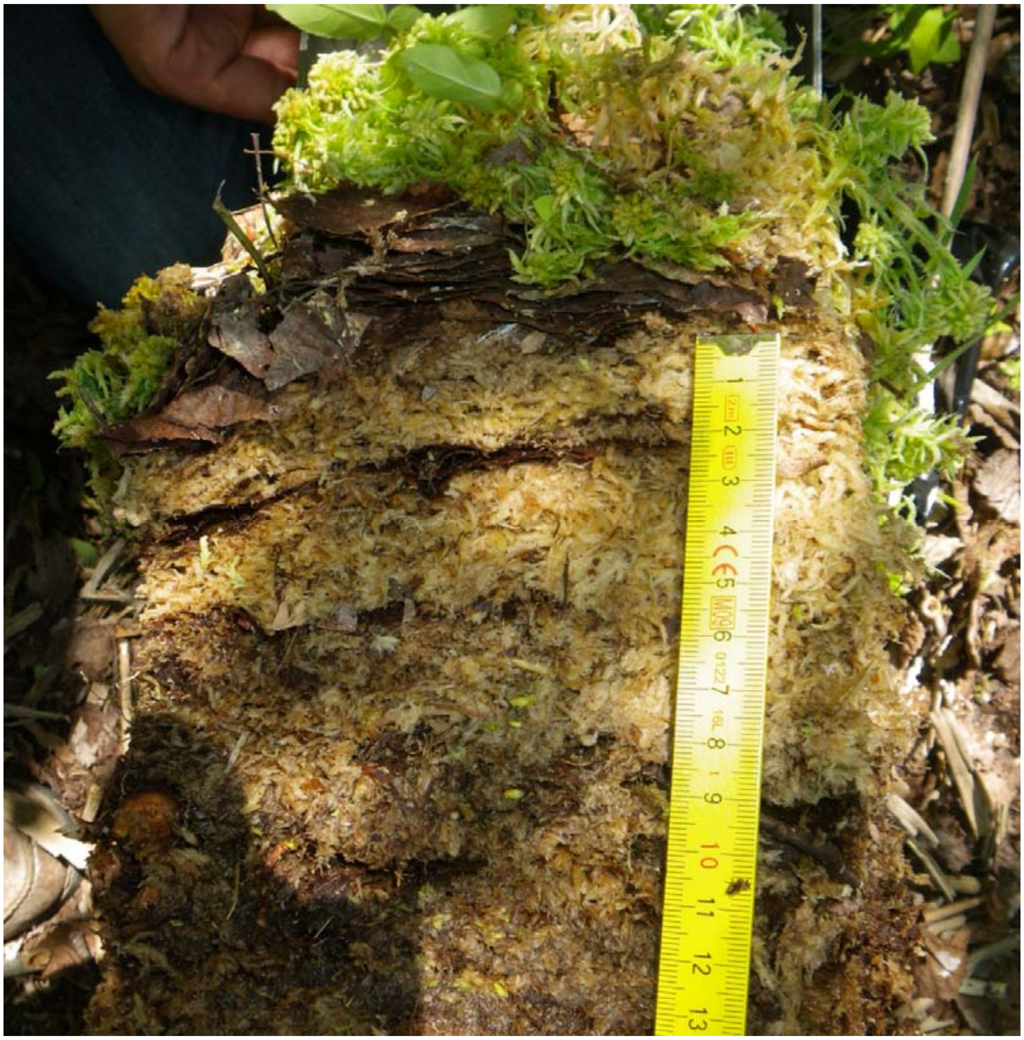

Supplementary Figure S6. The figure illustrates the peat accumulation rate in the top layers of the Posta Fibreno core (PF2) (photo by C.Z.). Here, as *Sphagnum* moss is spaced out by leaves of *Populus tremula* that annually fell off, the annual accumulation of peat can be measured, and reaches *ca*. 2 cm/yr.

8. Concentration of fallout radionuclides and ^{210}Pb core chronology

Supplementary Table S5. Fallout radionuclide (^{210}Pb, ^{226}Ra, ^{137}Cs) concentrations in selected samples throughout the top 200 cm of the peat profile.

Sample code	Average depth (cm)	AR (g/cm^2)	^{210}Pb Total (Bq/kg)	±	^{210}Pb Unsupported (Bq/kg)	±	^{210}Pb Supported † (Bq/kg)	±	^{137}Cs (Bq/kg)	±
PF2#01	-0.5	0.01	215	32	215	32	0	0	32	5
PF2#05	-4.6	0.11	334	33	332	33	2	6	35	5
PF2#09	-8.2	0.21	428	26	428	26	0	0	58	5
PF2#13	-11.2	0.30	175	26	174	27	1	6	50	5
PF2#17	-14.3	0.42	265	31	265	31	0	0	47	5
PF2#21	-18.0	0.58	306	29	306	29	0	0	46	5
PF2#32	-27.6	1.02	216	27	214	27	1	5	61	5
PF2#41	-35.6	1.33	181	29	179	29	2	6	399	9
PF2#44	-38.7	1.45	201	21	200	23	1	7	691	9
PF2#47	-42.1	1.59	214	21	214	21	0	0	205	5
PF2#53	-48.5	1.77	119	23	119	23	0	0	67	5
PF2#64	-60.3	2.11	108	23	107	23	1	5	128	5
PF2#72	-68.8	2.32	109	27	107	27	2	5	177	7
PF2#80	-75.1	2.47	139	29	138	30	2	6	184	6
PF2#84	-78.1	2.54	137	18	136	19	2	7	149	4
PF2#96	-89.1	2.81	108	31	108	31	0	0	99	7
PF B1#04	-101.5	3.09	123	63	121	63	2	6	205	12
PF B1#08	-109.7	3.30	109	40	109	40	0	0	220	8
PF B2#04	-152.2	4.16	50	13	49	13	2	3	36	2
PF B2#14	-173.2	5.12	42	13	42	13	0	0	31	2
PF B3#03	-200.3	6.44	27	8	26	8	2	2	21	2

† ^{226}Ra

The extremely rapid rate of peat accumulation required that a much greater number of peat samples than usual needed to be analysed. ^{241}Am was always below the minimum level of detection, thus providing an independent confirmation of the rapid rate of peat accumulation in the top 200 cm.

The ^{210}Pb inventory of the core is estimated to be at least 7,500 Bq/m^2 and corresponds to a mean ^{210}Pb supply rate of more than 230 Bq/m^2/y.

Raw ^{210}Pb dates calculated using the Constant Rate of Supply (CRS) model date 110 cm to 1968 and 39 cm to 1993, in both cases a little later than suggested by the ^{137}Cs record. Corrected

CRS model dates, calculated using the 1986 ^{137}Cs date as a reference point[49], give good agreement between the these two ^{137}Cs dates but date the second ^{137}Cs peak at 75 cm to 1974. A compromise that is reasonably consistent with all the data is to suppose that the peat layer at 75 cm is dated 1968 (Supplementary Table S6 and Fig. S7).

Supplementary Table S6. ^{210}Pb chronology of the top 200 cm of Posta Fibreno profile.

Sample code	Depth		Chronology			Sedimentation Rate		
	(cm)	(g cm^{-2})	Date (AD)	Age (y)	±	(g cm^{-2} y^{-1})	(cm y^{-1})	± (%)
	0.0	0.00	2012	0	0	-	-	-
PF2#01	-0.5	0.01	2012	0	1	0.060	2.4	16.6
PF2#05	-4.6	0.11	2011	1	2	0.061	2.3	12.4
PF2#09	-8.2	0.21	2009	3	2	0.065	2.2	9.8
PF2#13	-11.2	0.30	2007	5	2	0.060	1.7	17.3
PF2#17	-14.3	0.42	2006	6	2	0.056	1.4	14.2
PF2#21	-18.0	0.58	2003	9	2	0.055	1.3	13.1
PF2#32	-27.6	1.02	1994	18	2	0.053	1.2	17.1
PF2#41	-35.6	1.33	1988	24	3	0.051	1.2	21.2
PF2#44	-38.7	1.45	1986	26	3	0.052	1.4	18.2
PF2#47	-42.1	1.59	1983	29	3	0.057	1.8	18.8
PF2#53	-48.5	1.77	1980	32	3	0.060	2.1	22.3
PF2#64	-60.3	2.11	1975	37	3	0.061	2.3	25.0
PF2#72	-68.8	2.32	1972	40	3	0.066	2.5	29.0
PF2#80	-75.1	2.47	1969	44	4	0.082	3.4	26.0
PF2#84	-78.1	2.54	1968	44	4	0.109	4.6	20.2
PF2#96	-89.1	2.81	1966	46	4	0.150	6.3	32.9
PF B1#04	-101.5	3.09	1964	48	4	0.151	6.4	19.3
PF B1#08	-109.7	3.30	1963	49	4	0.110	4.1	19.3
PF B2#04	-152.2	4.16	1952	60	5	0.070	2.5	19.3
PF B2#14	-173.2	5.12	1938	74	7	0.070	1.4	19.3
PF B3#03	-200.3	6.44	1919	93	10	0.070	1.4	19.3

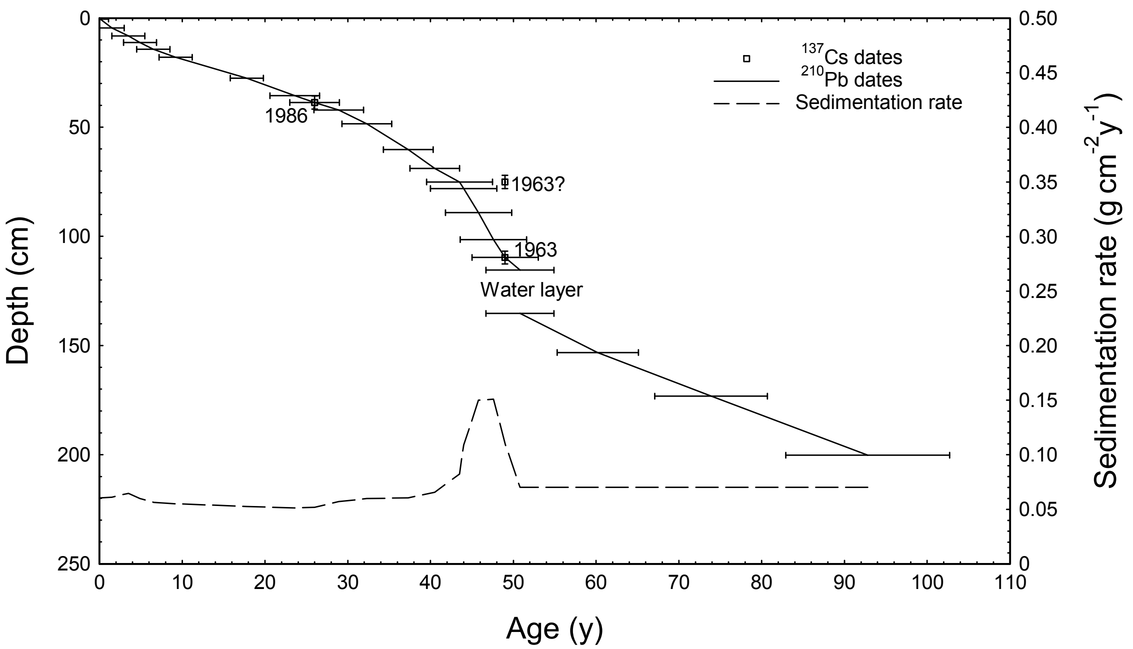

Supplementary Figure S7. Radiometric chronology of the Posta Fibreno peat profile showing the possible 1963 and 1986 depths determined from the ^{137}Cs stratigraphy, the ^{210}Pb dates as resulting from the CRS model (using the 1986 and 1963 ^{137}Cs dates as reference levels), and the sedimentation rates. More details are reported in Supplementary Table S6.

9. References

60. Boni, C., Bono, P. & Capelli, G. *Carta Idrogeologica del Territorio della Regione Lazio (Scala 1:250.000)*. (Regione Lazio, Università degli Studi "La Sapienza", Roma, 1988).

61. Agrillo, E., Bono, P., Casella, L., D'Andrea, L. & Fiori, D. in *Atti della prima giornata di studio "Tutela e conservazione dell'ecosistema acquatico Lago di Posta Fibreno area SIC/ZPS IT6050015"* (eds. Regione Lazio, R.N.R. Lago di Posta Fibreno, Agenzia Regionale Parchi) 55-81 (2008).

62. Mastrantuono, L., Di Vito, V. & Bazzanti, M. Spatial distribution of plant-associated invertebrates and environmental bioassessment in a natural riverine lake (Lake Fibreno, Central Italy). *J. Water Resource Prot.* **6**, 916-929 (2014).

63. Nisio, S., Caramanna, G. & Ciotoli, G. in *Natural and anthropogenic hazards in karst: Recognition, Analysis and Mitigation* (eds. M. Parise & J. Gunn) 23-45 (Geological Society London Special publications, 279, 2007).

64. Caramanna, G., Ciotoli, G. & Nisio, S. A review of natural sinkhole phenomena in Italian plain areas. *Nat. Hazards* **45**, 145-172 (2008).

65. Accordi, B. *et al.* Idrogeologia dell'alto bacino del Liri (Appennino centrale). Ricerche geologiche, climatiche, idrologiche, vegetazionali, geomorfologiche e sistematorie. *Geol. Rom.* **8**, 187-218 (1969).

66. Terracciano, S., Giordano, S., Bonini, I., Miserere, L. & Spagnuolo, V. Genetic variation and structure in endangered populations of Sphagnum palustre L. in Italy: a molecular approach to evaluate threats and survival ability. *Botany* **90**, 966-975 (2012).

67. Wardenaar, E. C. P. A new hand tool for cutting peat profiles. *Can. J. Bot. Rev. Can. Bot.* **65**, 1772-1773 (1987).

68. Jowsey, P. C. An improved peat sampler. *New Phytol.* **65**, 245–248 (1966).

69. Casella, L., Brugiapaglia, E., Shotyk, W., Miano, T. M. & Zaccone, C. Ecology and biogeography of the Posta Fibreno free-floating island. *J. Ecol.* (In preparation).

70. Daniels, R. E. & Eddy, A. *Handbook of European Sphagna*, 2nd Edn. (ed. Ecology, I. O. T., HMSO, London, 1990).

71. Sjörs, H. in *Ecosystems of the world, 4B. Mires: Swamp, bog, fen and moor* (ed. Gore A.J.P.) 69-94 (Elsevier, Amsterdam, 1983).

72. Brusa, G. The Sphagnum flora of the prealpine province of Varese, northern Italy. *Cryptogam. Bryol.* **21**, 257-265 (2000).

73. Fukuta, E., Sasaki, A. & Nakatsubo, T. Microclimate and production of peat moss Sphagnum palustre L. in the warm-temperate zone. *Plant Spec. Biol.* **27**, 110-118 (2012).

74. Wanner, H. *et al.* Mid- to Late Holocene climate change: an overview. *Quat. Sci. Rev.* **27**, 1791-1828 (2008).

75. Rydin, H. & Barber, K. E. Long-term and fine-scale coexistence of closely related species. *Folia Geobot.* **36**, 53-61 (2001).

76. Taylor, G. H. *et al. Organic Petrology*. (Gebrüder Borntraeger, Berlin, 1998).

77. Kuhry, P. & Vitt, D. H. Fossil carbon/nitrogen ratios as a measure of peat decomposition. *Ecology* **77**, 271-275 (1996).

78. Biester, H., Knorr, K.-H., Schellekens, J., Basler, A. & Hermanns, Y.-M. Comparison of different methods to determine the degree of peat decomposition in peat bogs. *Biogeosciences* **11**, 2691–2707 (2014).

79. Cocozza, C., D'Orazio, V., Miano, T. M. & Shotyk, W. Characterization of solid and aqueous phases of a peat bog profile using molecular fluorescence spectroscopy, ESR and FT-IR, and comparison with physical properties. *Org. Geochem.* **34**, 49-60 (2003).
